# Combing NLR, V20 and mean lung dose to predict radiation induced lung injury in patients with lung cancer treated with intensity modulated radiation therapy and chemotherapy

**DOI:** 10.18632/oncotarget.19032

**Published:** 2017-07-06

**Authors:** Wen-Yan Pan, Chao Bian, Guan-Lian Zou, Cui-Ying Zhang, Ping Hai, Ren Zhao, Yan-Yang Wang

**Affiliations:** ^1^ Department of Radiation Oncology, General Hospital of Ningxia Medical University, Yinchuan 750004, Ningxia, China; ^2^ Cancer Institute, Ningxia Medical University, Yinchuan 750004, Ningxia, China; ^3^ Graduate School, Ningxia Medical University, Yinchuan 750004, Ningxia, China

**Keywords:** neutrophil to lymphocyte ratio (NLR), V20, mean lung dose, radiation induced lung injury, lung cancer

## Abstract

The purpose was to evaluate the predictive value of baseline neutrophil to lymphocyte ratio (NLR) level in the incidence of grade 3 or higher radiation induced lung injury (RILI) for lung cancer patients. A retrospectively analysis with 166 lung cancer patients was performed. All of the enrolled patients received chemoradiotherapy at our hospital between April 2014 and May 2016. The Cox proportional hazard model was used to identify the potential risk factors for RILI. In this cohort, the incidence of grade 3 or higher RILI was 23.8%. Univariate analysis showed that radiation dose, volume at least received 20Gy (V20), mean lung dose and NLR were significantly associated with the incidence of grade 3 or higher RILI (*P* = 0.012, 0.008, 0.012, and 0.039, respectively). Multivariate analysis revealed that total dose ≥ 60 Gy, V20 ≥ 20%, mean lung dose ≥ 12 Gy, and NLR ≥ 2.2 were still independent predictive factors for RILI (*P* = 0.010, 0.043, 0.028, and 0.015, respectively). A predictive model of RILI based on the identified risk factors was established using receiver operator characteristic curves. The results demonstrated that the combination analysis of V20, mean lung dose and NLR was superior to either of the variables alone. Additionally, we found that the constraint of V20 and mean lung dose were meaningful for patients with higher baseline NLR level. If the value of V20 and mean lung dose lower than the threshold value, the incidence of grade 3 or higher RILI for the high NLR level patients could be decreased from 63.3% to 8.7%. Our study showed that radiation dose, V20, mean lung dose and NLR were independent predictors for RILI. Combination analysis of V20, mean lung dose and NLR may provide a more accurate model for RILI prediction.

## INTRODUCTION

As one of dose-limiting factors, radiation induced lung injury (RILI) limits the usage of radiotherapy in lung cancer patients. Although a growing body of evidence has shown that some clinical factors, dosemetric factors and biology factors are associated with the incidence of RILI, there is no consensus about the prediction of RILI in patients with lung cancer until now [1–4]. Therefore, accurately assess individual patient's risk of developing RILI deserves further investigation.

More and more studies suggest that the inflammation background of host had an influence on the incidence of RILI [5–7]. The neutrophil to lymphocyte ratio (NLR), which involves measurement of a subgroup of white blood cells, has been identified as a marker of systemic inflammation. Increased pre-treatment NLR level has been used in combination with other inflammatory markers to determine the prognosis of many diseases [8–12]. However, as we known, the relationship between pre-radiotherapy NLR level and RILI has not been well documented before.

In this study, we investigated the prediction role of NLR and other clinical or dosimetric risk factors for grade 3 or higher RILI in 166 patients with lung cancer. All of the enrolled patients received concurrent or sequential chemoradiotherapy in our department. The combination value of NLR and other meaningful factors in the prediction of grade 3 or higher RILI was also determined.

## RESULTS

The features of the final analyzed patients, tumors, and treatments are list in Table 1. There were 116 men and 35 women, with median age of 60 years (range, 29 to 78 years). Among them, 50.3% were non-small cell lung cancer (NSCLC), 10.6% received surgery treatment. Of these 151 patients, 72 received sequential chemoradiotherapy, 79 received concurrent chemoradiotherapy. 57.6% of these patients received 50 to 60 Gy of radiation, and 42.4% received equal or more than 60 Gy. The median chemotherapy cycles of the whole study population was 4 (range, 1 to 10 cycles). In total, 36 patients (23.8%) developed grade 3 or higher RILI, including two patients died of RILI. These events was frequently observed within one to three months after radiotherapy.

**Table 1:** Clinicopathologic and dosimetric factors of enrolled lung cancer patients according to different NLR level

Characteristics	NLR, (*n*)		*P* value
	≥ 2.2	< 2.2	
Age, years			
≥ 60	36	43	
< 60	40	32	0.255
Sex			
Male	60	56	
Female	16	19	0.567
KPS			
90	33	51	
70, 80	43	24	0.003
Smoke			
Ever	51	42	
Never	25	33	0.183
COPD			
Yes	10	8	
No	66	67	0.803
Histology			
NSCLC	40	36	
SCLC	36	39	0.627
TNM stage			
I–II	40	35	
III	36	40	0.517
PORT			
Yes	11	15	
No	65	60	0.396
Dose			
≥ 60 Gy	32	32	
50–60 Gy	44	43	1.000
V20			
≥ 20%	42	53	
< 20%	34	22	0.064
MLD			
≥ 12 Gy	40	34	
< 12 Gy	36	41	0.417
Neoadjuvant CT			
Yes	61	54	
No	15	21	0.257
Concurrent CT			
Yes	38	41	
No	38	34	0.626
Adjuvant CT			
Yes	35	25	
No	41	50	0.135

Abbreviations: NLR, neutrophil to lymphocyte ratio; KPS, Karnofsky scores; COPD, chronic obstructive pulmonary disease; NSCLC, non-small cell lung cancer; SCLC, small cell lung cancer; PORT, post-operative radiotherapy; V20, volume at least received 20Gy; MLD, mean lung dose; CT, chemotherapy.

On univariate analysis, total dose, the volume at least received 20Gy (V20), mean lung dose (MLD) and NLR were significantly associated with grade 3 or higher RILI (*P* = 0.012, 0.008, 0.012, and 0.039, respectively). These four meaningful factors were then entered into the multivariate analysis. The results of the multivariate analysis revealed that total dose ≥ 60 Gy, V20 ≥ 20%, MLD ≥ 12 Gy, and NLR ≥ 2.2 were still independent predictive factors for grade 3 or higher RILI (*P* = 0.010, 0.043, 0.028, and 0.015, respectively). The results of the univariate and multivariate analysis are shown in Table 2.

**Table 2:** Univariate and multivariate analysis of clinicopathologic and dosimetric factors associated with the incidence of grade 3 or higher RILI

Clinicopathologic and dosimetric factors		Univariable analysis			Multivariable analysis		
		Hazard ratio	95% CI	*P*-value	Hazard ratio	95% CI	*P*-value
Age, years	< 60 vs. ≥ 60	0.987	0.499–1.954	0.971			
Gender	Female vs. Male	0.685	0.283–1.660	0.403			
KPS	90 vs. 80 or 70	0.790	0.393–1.588	0.508			
Smoke	Never vs. Ever	1.199	0.601–2.391	0.607			
COPD	No vs. Yes	0.517	0.213–1.253	0.144			
Histology	NSCLC vs. SCLC	1.839	1.905–3.739	0.092			
TNM stage	I–II vs. III	0.689	0.345–1.374	0.290			
PORT	Yes vs. No	0.623	0.281–1.381	0.244			
Dose	≥ 60 Gy vs. 50–60 Gy	2.495	1.227–5.074	0.012	2.548	1.248–5.202	0.010
V20	≥ 20% vs. < 20%	3.617	1.396–9.371	0.008	2.865	1.033–7.944	0.043
MLD	≥ 12 Gy vs. < 12 Gy	3.835	1.664–8.841	0.002	2.728	1.116–6.666	0.028
NLR	< 2.2 vs. ≥ 2.2	0.466	0.226–0.962	0.039	0.406	0.196–0.839	0.015
Neoadjuvant CT	Yes vs. No	1.269	0.524–3.073	0.598			
Concurrent CT	Yes vs. No	1.084	0.546–2.150	0.818			
Adjuvant CT	Yes vs. No	0.911	0.457–1.818	0.792			

Abbreviations: RILI, radiation induced lung injury; CI, confidence interval; KPS, Karnofsky scores; COPD, chronic obstructive pulmonary disease; NSCLC, non-small cell lung cancer; SCLC, small cell lung cancer; PORT, post-operative radiotherapy; V20, volume at least received 20Gy; MLD, mean lung dose; NLR, neutrophil to lymphocyte ratio; CT, chemotherapy.

**Figure 1:**
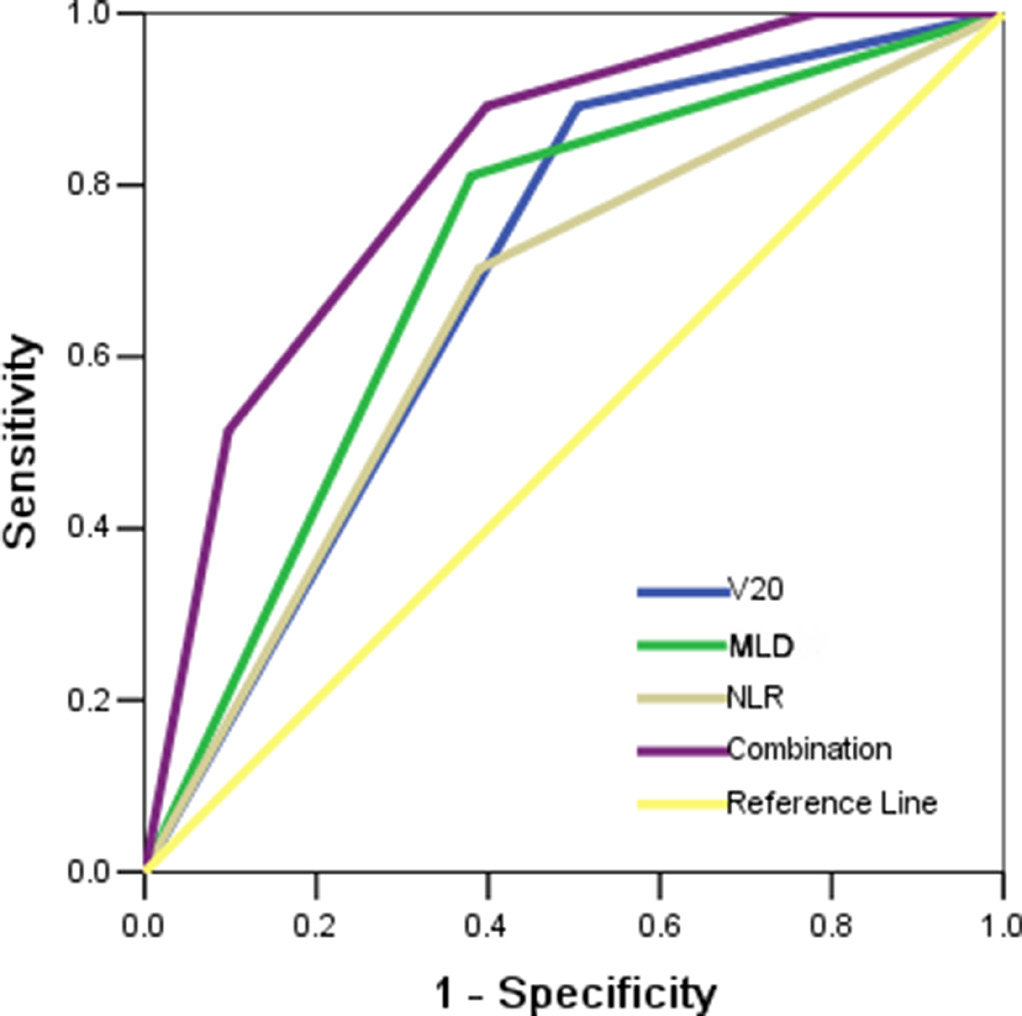
Receiving operator characteristic curve based on the sensitivity and specificity of V20 alone, MLD alone, NLR alone, or all of these three factors combined

In order to establish the predictive model of grade 3 or higher RILI, receiver operating characteristic (ROC) curves were generated for V20, or MLD, or NLR, or the combination of these three factors. The results demonstrated that these three parameters alone were poor in predicting grade 3 or higher RILI. The area under the curve (AUC) values for V20, MLD, and NLR were 0.69 (95% CI, 0.60–0.78, *P <* 0.001), 0.72 (95% CI, 0.62–0.81, *P <* 0.001), and 0.66 (95% CI, 0.56–0.76, *P* = 0.004), respectively. However, combining all three parameters into a single model improved the predictive ability compared to either of the variables alone, producing an AUC of 0.82 (95% CI, 0.74–0.89, *P <* 0.001) (Figure 1).

The benefit of combining analysis of these three meaningful parameters was explored further by using threshold values, which determined by ROC curves. The results showed that the threshold values of V20, MLD, and NLR was 20%, 12Gy, and 2.2, respectively. Using threshold values for V20 and MLD, patients with high NLR level were stratified into 4 subgroups: high V20 and high MLD (group 1), low V20 and high MLD, (group 2), high V20 and low MLD (group 3), and low V20 and low MLD (group 4). The incidence of grade 3 or higher RILI was 63.3%, 40.0%, 25.0%, and 8.7% in group 1 to 4, respectively. The results revealed that the threshold values for V20 and MLD were meaningful for the reduction of probability of grade 3 or higher RILI, especially for the high pre-treatment NLR level lung cancer patients.

## DISCUSSION

In current study, an incidence of 23.8% was observed for the grade 3 or higher RILI, which is similar to the previous study [13]. And the risk factors for the development of RILI were also assessed in this study. No clinical factors, except for NLR, was found significantly associated with RILI. In regard to the dosimetric factors, radiation dose, V20, and MLD were found to be significantly associated with grade 3 or higher RILI in present study. To improve the predictive ability of RILI, the combined analysis of V20, MLD, and NLR was performed using ROC model. For the determination of total prescription dose was usually depend on the histology or the combined therapy method, it did not entered into the ROC analysis. The results of ROC model demonstrated that combining these three factors improved the predictive ability compared to that with either of the factors alone. Subsequently, we found that the constraint of V20 and MLD were meaningful for the higher baseline NLR level patients. If the value of V20 and MLD lower than the threshold value, the incidence of grade 3 or higher RILI for the high NLR level patients can be decreased from 63.3% to 8.7%.

The role of dose-volume parameters in predicting RILI were suggested in several previous studies [14–17]. Some dosimetric parameters, including V20 and MLD, were reported had a relationship with the occurrence of RILI. However, the consensus thresholds of these parameters did not draw definitive conclusions [17]. In order to reduce the risk of RILI, V20 should be limit less than 30–35%, and MLD should be limit less than 20–23 Gy, which recommended by Quantitative Analysis of Normal Tissue Effects in the Clinic (QUANTEC) [17]. In current study, we found that the threshold values of V20 and MLD was 20% and 12Gy, which is lower than the recommendation of QUANTEC. The reasons of this phenomenon not only include heterogeneity of different study population, but also depend on the radiation therapy techniques and the combined treatment approaches. In current study, all of the patients received intensity modulated radiation therapy (IMRT) and more neoadjuvant chemotherapy patients were enrolled.

Since the dose-volume parameters were population based predictors for RILI, more individualized predictors of RILI are still needed. In current study, we revealed that pre-treatment NLR level, a representative indicator of systemic inflammation, was associated with grade 3 or higher RILI. However, the exact mechanism underlying this effect remains unclear. The most possible reason is the host inflammatory response participates the initiation and progression of RILI [5–7]. As we known, neutrophils could secrete cytokines and chemokines mediate inflammatory cell recruitment and angiogenesis. In addition, an elevated neutrophil could suppress the cytolytic activity of lymphocytes, natural killer cells, activated T cells, and adaptive immune response suppression [18, 19]. On the other side, lymphocytes exert a critical role in cytotoxic cell death and cytokine production that reduce inflammation infiltration [20, 21]. Some preclinical studies showed that decreasing the amount of neutrophils or macrophages could reduce the amount of lung fibrosis [7, 22, 23]. And these results were also confirmed in clinical setting for the first time in current study. Transforming growth factor beta 1 (TGF-β1) is a multifunctional growth factor and exerts a critical role in the development of RILI [24–26]. However, many mechanisms on the interaction between neutrophil and TGF-β1 have been found [27, 28]. The interaction between these two factors may also contribute to the interpretation of the role of NLR in RILI prediction.

As a retrospective study, there are a couple of limitations. Firstly, the bias of patient selection and the diagnosis of RILI which based on the medical records may have an impact on the interpretation of the results. Secondly, although we confirmed the predictive value of NLR in this study, the optimal cut-off value of NLR still need more research to establish. Thirdly, poor pulmonary function was thought to be an important patient-related risk factor for the development of RILI [29], nevertheless, pulmonary function tests parameters, such as forced expiratory volume, had not been evaluated in current study for the missing data of some patients. Lastly, the relationship between NLR and other predictive biological markers, such as TGF-β1 and interferons (IFNs), interleukin (IL)-6, IL-1, IL-10, and tumor necrosis factor (TNF)-α, was not assessed. The combination of these biological markers with NLR may further improve the prediction of RILI.

In conclusion, we demonstrated that total dose ≥ 60 Gy, V20 ≥ 20%, MLD ≥ 12 Gy, and NLR ≥ 2.2 were independent predictors for the occurrence of grade 3 or higher RILI of lung cancer patients who received IMRT and chemotherapy. And the combined analysis of these factors could improve the predictive ability of RILI, thereby, affording the opportunity to individualize therapy. However, in order to optimal use these easy accessed factors in daily practice, validation in a prospective multicenter study is essential.

## MATERIALS AND METHODS

### Patient population

166 lung cancer patients were enrolled in this study. All of the enrolled patients received sequential or concurrent chemoradiotherapy at our hospital between April 2014 and May 2016. 15 patients with coexistent obstructive pneumonia were excluded from the analysis. This investigation was approved by The General Hospital of Ningxia Medical University institutional review board (2016–200).

### Treatment

All patients underwent planning computed tomography (CT) using a Somatom Sensation Open CT scanner (Simens Medical Systems, Munich, Germany). Axial CT images were obtained with a maximum slice separation of 0.5 cm from the mandible to the lower edge of the liver in the treatment position. The image sets were transferred to the Pinnacle V8.0 treatment planning system (Philips Medical, Madison, WI, USA) for contouring and planning. Following the RTOG recommendations, the target volume and organs at risk structures were contoured on individual simulation CT slices. All contouring was carried out by one radiation oncologist and verified by a second radiation oncologist.

All the irradiation was given using the IMRT technique with tissue inhomogeneity corrections. The collapsed cone algorithm (CC convolution) was used for the final plan calculation and to determine all dose-volume histogram (DVH) values. The prescription dose for the definitive treatment was 60–64 Gy/30–32 fractions, for the postoperative radiotherapy was 50Gy/25 fractions. The criterion for acceptance of the plan was that at least 99% of the planning target volume (PTV) was covered by 95% of the prescription dose and the maximum dose was less than or equal to 105%. Normal tissue dose-volume constraints were applied. Limited volumes of spinal cord were allowed to exceed 45 Gy. The mean dose of heart were constrained from receiving more than 35 Gy. The mean dose of esophagus was kept from exceeding 34 Gy. The MLD was kept from exceeding 15 Gy, and V20 was kept less than 20% or lower if possible. The total normal lung volume was defined as the volume of both lungs minus the gross target volume (GTV).

The most used regimens for concurrent chemoradiotherapy consisted of vinorelbine and cisplatin for NSCLC, etoposide and cisplatin for small cell lung cancer (SCLC).

### Follow-up

As the primary endpoint of this study, grade 3 or higher RILI was classified according to Common Terminology Criteria for Adverse Events version 3.0. Patients were assessed weekly during whole radiotherapy procedure. After radiation, all patients were subjected to a follow-up every 2 to 4 weeks for up to 6 months, and then every 3 months for 2 years. History and physical examination, chest CT scan were checked at each follow-up. RILI was diagnosed according to clinical symptoms, laboratory test results, chest CT scans. The times to endpoints development were calculated from the beginning of radiotherapy; patients not experiencing the endpoint were censored at the last follow-up. The median follow-up time was 15 months (range, 1 to 31 months).

### Statistical analysis

The ROC curve was used to identify the best cut-off points for different variables with which to assess the risk of RILI. The AUC was used to assess the predictive value of each risk factor. Differences in clinical factors and DVH metrics between the higher and lower NLR groups were assessed by a chi-square test or Fisher's exact test where appropriate. Cox proportional hazards analysis was used to calculate the hazard ratio (HR) and confidence interval (CI) to evaluate the influence of clinicopathological and dosimetric variables on the time of RILI development. The analysis performed at the end of follow-up. The meaningful factors which confirmed in univariate analysis were then tested by multivariate analysis. All *P* values of less than 0.05 were considered statistically significant. Statistical analysis was performed using SPSS 16.0 statistical software package (SPSS Inc, Chicago, IL).
